# Cerclage position, cervical length and preterm delivery in women undergoing ultrasound indicated cervical cerclage: A retrospective cohort study

**DOI:** 10.1371/journal.pone.0178072

**Published:** 2017-06-01

**Authors:** Joanna R. Cook, Susan Chatfield, Manju Chandiramani, Lindsay Kindinger, Stefano Cacciatore, Lynne Sykes, Tiong Teoh, Andrew Shennan, Vasso Terzidou, Phillip R. Bennett

**Affiliations:** 1 Parturition Research Group, Imperial College London, Institute of Reproductive and Developmental Biology, Hammersmith Hospital Campus, Du Cane Road, London, United Kingdom; 2 Women's Health Academic Centre, King’s Health Partners, King’s College, St Thomas' Hospital Campus, Westminster Bridge Road, London, United Kingdom; University of Vermont, UNITED STATES

## Abstract

**Objective:**

The objectives were to assess whether anatomical location of ultrasound (USS) indicated cervical cerclage and/or the degree of cervical shortening (cervical length; CL) prior to and following cerclage affects the risk of preterm birth (PTB).

**Method:**

A retrospective cohort study of 179 women receiving cerclage for short cervix (≤25mm) was performed. Demographic data, CL before and after cerclage insertion, height of cerclage (distance from external os) and gestation at delivery were collected. Relative risk (RR) and odds ratio (OR) of preterm delivery were calculated according to the anatomical location of the cerclage within the cervix and the CL before and after cerclage as categorical and continuous variables. Partition tree analysis was used to identify the threshold cerclage height that best predicts PTB.

**Results:**

25% (n = 45) delivered <34 weeks and 36% (n = 65) delivered <37 weeks. Risk of PTB was greater with cerclage in the distal 10mm (RR2.37, 95% CI 1.45–3.87) or the distal half of a closed cervix (RR2.16, 95% CI 1.45–3.87). Increasing absolute cerclage height was associated with a reduction in PTB (OR 0.87, 95% CI 0.82–0.94). A cerclage height <14.5 mm best predicts PTB (70.8%). Increasing CL following cerclage was associated with a reduction in PTB (OR0.87, 95% CI 0.82–0.94). Conversely, the risk of PTB was increased where CL remained static or shortened further following cerclage (RR2.34, 95% CI 1.04–5.25).

**Conclusion:**

The higher a cerclage was placed within a shortened cervix, the lower the subsequent odds of PTB. Women whose cerclage is placed in the distal 10mm of closed cervix or whose cervix fails to elongate subsequently, should remain under close surveillance as they have the highest risk of PTB.

## Introduction

Preterm birth (PTB) is a syndrome with multiple aetiologies including infection/inflammation, abruption, maternal and fetal stress and cervical weakness. The incidence of true cervical insufficiency is about 1% of pregnant women [1]. Cervical cerclage is commonly used to prevent or delay PTB in women with a history suggestive of cervical insufficiency or a short cervix on ultrasound scan (USS) [2]. Cerclage may be placed in three different clinical scenarios: following multiple mid-trimester losses (MTL) or preterm delivery (history indicated cerclage), when the cervix is dilating in the absence of contractions (rescue cerclage) or when the cervix shortens (usually defined as ≤ 25 mm) in women with a history of cervical surgery or PTB (USS indicated cerclage). The efficacy of USS indicated cerclage compared with conservative management has been assessed with mixed results [3–5]. The variability in observed outcomes may be secondary to variation in the anatomical placement of the cerclage within the cervix. It is generally assumed that the closer a cerclage is placed to the internal cervical os the more effective it will be, but this has not been proven [6]. Some surgeons consider that in the McDonald technique it is acceptable to place the cerclage in the mid or lower portion of the cervix. We sought to examine the outcomes of women undergoing USS indicated cerclage relative to the anatomical placement of the suture within the cervix. We examined both the distance of the cerclage from the external os (cerclage height), and cerclage position defined as a proportion of total cervical length (CL). We also assessed if CL prior to and after insertion of the cerclage, or if a change in CL following cerclage insertion, was predictive of PTB.

## Methods

### Data collection

Data were collected from women attending specialist prematurity antenatal clinics in two UK tertiary referral centres between 2001 and 2012. Women were referred for trans-vaginal (TV) USS cervical surveillance when they had a history of PTB (defined as delivery at 24+0 to 36+6 weeks gestation), mid-trimester loss (defined as miscarriage at 14+0 to 23+6 weeks gestation), or cervical treatment (knife cone biopsy or large loop excision of the transformation zone following abnormal cervical cytology). A retrospective cohort study of women with a singleton pregnancy undergoing USS-indicated cerclage was performed. USS-indicated cerclage was defined as a suture being placed in the index pregnancy when CL was ≤ 25 mm and the external os remained closed. Women received either nylon or mersilene sutures. All women were re-scanned within 14 days of cerclage placement.

Demographic data, CL before and after insertion of cerclage and distance of cerclage from external cervical os, were collated. Outcome measures were delivery prior to 34 and 37 completed weeks gestation. This study was exempt from requiring ethical approval under the UK Health and Social Care Act 2011 which states that research involving anonymised clinical data is excluded from research ethics committee review. Data were anonymised by JC and SC (at Queen Charlotte’s and Chelsea Hospital) and MC and SC (at St. Thomas’ Hospital). The authors interacted with the patients when they performed the ultrasound scans or inserted cervical cerclages.

The standardised method of obtaining CL as described by Berghella and recommended by the Cervical Length Education and Review (CLEAR) programme (Perinatal Quality Foundation, OK, USA) was used [7, 8]. The woman was asked to empty her bladder and then the probe was inserted into the anterior fornix of the vagina to obtain a sagittal long-axis view of the cervical canal. Care was taken not to exert pressure on the cervix and inadvertently elongate it. The CL was measured from the internal to the external os and the distance between the cervical suture (if inserted) and the internal os was also measured. At least three measurements were obtained for each dimension and the shortest was used in the analysis. One of two senior obstetricians (AS, PB) inserted all cerclages using either the McDonald or modified Shirodkar technique where the bladder is dissected from the anterior cervix and the suture ‘buried’ underneath. The latter technique was used where examination under anaesthetic suggested that bladder dissection would be needed to ensure satisfactory placement of the stitch.

### Data analysis

The cohort was initially categorically divided according to distance of cerclage in millimetres (mm) from the external os (cerclage height) at first USS after suture insertion. The relative risks (RR) of PTB at cerclage heights of <10mm and <15mm, relative to the remainder of the cohort, were calculated. Cerclage height was also analysed as a continuous variable and association with subsequent delivery at <34 and <37 weeks gestation was assessed using logistic regression. A decision tree analysis [9] was also performed to select the optimal cerclage height threshold to predict PTB <34 weeks and <37 weeks gestation. Partition tree analysis was completed using R package “rpart”: function “rpart.”

Examining absolute cerclage height does not take account of the range of CLs in the cohort, such that poorer outcomes in women with sutures placed <10 mm from the external os may merely reflect the presence of an already shorter cervix, and thus an increased risk of preterm delivery due to reduced initial total CL. In order to take account of the different CLs within the cohort, we examined the gestation at delivery according to cerclage height as a percentage of total CL, as observed at the first USS (<14 days) after suture insertion. The RR of preterm delivery was therefore calculated in those with a cerclage in the distal half of the cervix compared with those with sutures in the upper half of the cervix. Cerclage height as a percentage of CL was also assessed as a continuous variable using logistic regression.

We further hypothesised that women with more distally placed sutures may have a greater RR of preterm delivery because a shorter cerclage height reflects a smaller initial CL. We therefore assessed gestational age at delivery in women relative to their CL at the time that a decision for cervical cerclage was made, and compared outcomes in those with initial CLs ≤10 mm and 11–20 mm with those women with the ‘longest’ CLs in our cohort (21–25 mm). Initial CL was also assessed as a continuous variable using logistic regression. Furthermore, in a previous observational study undertaken by our group, we demonstrated that CL increases following cerclage insertion (mean increase 11.5 mm two weeks post-insertion, 95% CI 5.9–17.1; p<0.001) [10]. We therefore examined CL at the first TV USS after cerclage insertion and compared outcomes in those whose CL remained ≤25 mm with those whose CL ‘normalised’ to >25mm following cerclage, we also assessed post-stitch CL as a continuous variable. We then compared outcomes in women whose CL did not change or shortened following cerclage with those whose CL increased by any amount, and also assessed if ‘change in CL’ as a continuous variable was associated with PTB. Statistical analysis was carried out using STATA v11 (Texas, USA) unless otherwise stated.

## Results

Two hundred and eleven women undergoing USS-indicated cerclage were identified. Thirty-two cases were excluded because of incomplete USS data, unknown obstetric outcomes or the insertion of a second cerclage. This resulted in a total cohort of 179 women for analysis.

The median maternal age was 32 years (range 18–48 years). Forty-seven percent (n = 79) of the cohort were Black, 37% (n = 66) were White, 11% (n = 20) were Asian and in 8% (n = 14), the ethnicity was unrecorded. Thirty-five percent (n = 62) had a history of preterm delivery, 53% (n = 94) had had a previous MTL and 26% (n = 47) had received earlier cervical treatment including large loop excision of the transformation zone or knife cone biopsy (some women had more than one risk factor). Women received a TV USS indicated cerclage at a median of 18 weeks gestation (range 11–24 weeks). Although USS indicated cerclage is most often placed at >16 weeks, if cervical shortening was demonstrated prior to this gestation, a cerclage was still offered following aneuploidy screening.

In the index pregnancy, 25% (n = 45) of women delivered prior to 34 weeks gestation (range 19–34 weeks) and 36% (n = 65) delivered prior to 37 (range 19–37) weeks gestation, highlighting that this cohort overall was at high risk of preterm birth. In women with a previous PTB or MTL (n = 136), 26% (n = 36) delivered prior to 34 weeks gestation and 40% (n = 54) delivered before 37 weeks, indicating that this sub-group was at greatest risk of delivering preterm. In women who had only had previous cervical surgery (n = 43) (ie never had a previous PTB/MTL), 21% (n = 9) delivered prior to 34 weeks gestation and 26% (n = 11) delivered before 37 weeks.

### 1. Absolute cerclage height (mm)

The distance between the cerclage and the external os in mm (cerclage height) at first USS after suture insertion ranged from 2-28mm (median 15mm). ‘Cerclage height’ was initially treated as a dichotomous variable and the cohort was divided into those whose cerclage was placed within 10 mm of the external os (n = 47) and those whose cerclage was >10 mm from the external os (n = 132). Preterm delivery at <34 weeks gestation occurred in 27 (20%) women with a cerclage >10mm from the external os and in 18 (38%) women with a cerclage placed ≤10 mm from the external os. Preterm delivery at <37 weeks gestation occurred in 38 (29%) and 27 (57%) of women with cerclage placed above and below 10mm from the external os respectively. The cohort was then divided into those whose cerclages were placed within 15 mm of the external os (n = 106) and those with cerclages >15 mm from the external os (n = 73). The RR of PTB at different cerclage heights, relative to the remainder of the cohort, was calculated (Table 1). The risk of PTB was highest in those women where the suture is placed most superficially.

**Table 1:** Risk of preterm birth by height of cerclage from external os. The relative risk of preterm birth before 34 and 37 weeks of gestation is shown in subgroups of women depending on the distance between external os and suture in mm compared to the remainder of the cohort. The relative risk of preterm birth is increased in those with a lower cerclage height.

Gestation at delivery	Women with cerclage height >10mm (n = 132)	Women with cerclage height ≤10mm (n = 47)	RR (95% CI)	Women with cerclage height >15mm (n = 73)	Women with cerclage height ≤15 mm (n = 106)	RR (95% CI)
<34 weeks n (%)	27 (20%)	18 (38%)	1.85 (1.14–2.99) p = 0.0155	9 (12%)	36 (34%)	1.53 (1.23–1.90) p = 0.0010
<37 weeks n (%)	38 (29%)	27 (57%)	2.37 (1.45–3.87) p = 0.0005	14 (19%)	51 (48%)	1.63 (1.29–2.04) p = 0.0001

Absolute cerclage height was also assessed as a continuous variable using logistic regression analysis. Increasing cerclage height reduced the odds of PTB <34 weeks (Odds ratio (OR) 0.90, 95% CI 0.84–0.96) and <37 weeks (OR 0.87, 95% CI 0.82–0.94).

A decision tree analysis was also performed to select the best cerclage height cut-off to predict PTB <34 weeks and <37 weeks. A cerclage height <13.5 mm at first USS scan following insertion best predicted PTB <34 weeks (64.4%), whilst a cerclage height <14.5 mm best predicted PTB <37 weeks (70.8%).

### 2. Cerclage height as a percentage of total cervical length

Cerclage height as a percentage of total CL was initially treated as a dichotomous variable and the RR of preterm delivery in those women with a cerclage placed in the distal half of the cervix (n = 58) relative to those with cerclage placed in the proximal half of the cervix (n = 121) was calculated (Table 2). The results show that the risk of preterm delivery was increased when the suture was placed in the distal half of the cervix.

**Table 2:** Risk of preterm birth by height of cerclage as a percentage of cervical length. The relative risk of preterm birth before 34 and 37 weeks of gestation is shown in women with cerclages placed in the distal half (represented as < 50% height) of the cervix relative to those with cerclages placed in the proximal half (represented as > 50%).

Gestation at delivery	Women with cerclage height ≥50% of cervical length (n = 121)	Women with cerclage height <50% of cervical length (n = 58)	RR (95% CI)
<34 weeks n (%)	23 (19%)	22 (38%)	1.82 (1.21–2.74) p = 0.0063
<37 weeks n (%)	33 (27%)	32 (55%)	2.16 (1.42–3.28) p = 0.0003

Cerclage height as a percentage of total CL was also assessed as a continuous variable using logistic regression analysis. Increasing cerclage height as a percentage of total CL was not associated with a reduction in the odds of delivering preterm at <34 weeks (OR 0.99, 95% CI 0.97–1.01), or at <37 weeks (OR 0.98, 95% CI 0.97–1.00), suggesting either a threshold effect in optimal stitch positioning or that those with a very short cervix still had greater risk of PTB, even with a higher stitch.

### 3. Initial absolute cervical length

CL at the time that a decision for cervical cerclage was made ranged from 1 – 25mm (median 17mm). Women with the most extreme shortening of CL (<10mm) had the highest incidences of PTB (31% <34 weeks, 43% <37 weeks) and the risk of PTB <37 weeks was higher than those with ‘borderline short’ CLs of 21–25 mm (RR 1.63, 95% CI 1.04–2.56), however, RR did not reach significance for delivery prior to 34 weeks (Table 3). This effect was also observed in women with moderate cervical shortening to 11–20 mm who had a higher rate of PTB (26% <34 weeks, 39% <37 weeks) than those with mild cervical shortening of 21–25 mm and a higher risk of delivery <37 weeks (RR 1.23, 95% CI 1.03–1.48) (Table 3).

**Table 3:** Risk of preterm birth by cervical length at time of decision for cerclage. The relative risk of preterm birth before 34 and 37 weeks of gestation is shown in subgroups of women depending on the cervical length (in mm) at the time of decision for cerclage, compared with those with the ‘longer’ cervical lengths (21-25mm).

Gestation at delivery	Number of women with cervical length 21-25mm (n = 38)	Number of women with cervical length 11-20mm (n = 106)	RR (95% CI)	Number of women with cervical length ≤10mm (n = 35)	RR (95% CI)
<34 weeks n (%)	6 (16%)	28 (26%)	1.16 (0.95–1.41) p = 0.19	11 (31%)	1.51 (0.95–2.40) p = 0.11
<37 weeks n (%)	8 (21%)	42 (39%)	1.23 (1.03–1.48) p = 0.039	15 (43%)	1.63 (1.04–2.56) p = 0.045

When CL at decision for cerclage was treated as a continuous variable it was found that increasing CL reduced the odds of preterm delivery prior to 34 weeks (OR 0.94, 95% CI 0.87–0.99) and prior to 37 weeks (OR 0.94, 95% CI 0.89–0.99) however, these findings only just reach significance.

### 4. Post cerclage cervical length

The absolute CL at the first TV USS after cerclage insertion ranged from 5–45 mm (median 27 mm). In women whom the post-cerclage CL remained ≤25 mm (n = 79), 33% delivered prior to 34 weeks and 44% prior to 37 weeks gestation. There was an increased RR of delivering prior to 34 weeks (RR 1.46, 95% CI 1.05–2.02) and prior to 37 weeks (RR 1.40, 95% CI 1.01–1.93) compared with those whose cervix returned to a normal length (>25 mm) (Table 4).

**Table 4:** Risk of preterm birth by cervical length after cerclage insertion. The relative risk of preterm birth before 34 and 37 weeks of gestation is shown in women who still had a short cervix (≤25mm) after cerclage insertion, relative to those whose cervical length increased to >25mm post procedure.

Gestation at delivery	Number of women with cervical length >25mm (n = 100)	Number of women with cervical length ≤25mm (n = 79)	RR (95% CI)
<34 weeks n (%)	19 (19%)	26 (33%)	1.46 (1.05–2.02) p = 0.033
<37 weeks n (%)	30 (30%)	35 (44%)	1.40 (1.01–1.93) p = 0.048

When CL following cerclage was treated as a continuous variable it was found that increasing CL reduced the odds of preterm delivery prior to 34 weeks (OR 0.94, 95% CI 0.90–0.99), and prior to 37 weeks (OR 0.93, 95% CI 0.89–0.98).

### 5. Change in cervical length (mm) after cerclage insertion

Eighty-eight % (n = 158) of women exhibited some lengthening of the cervix at the first TV USS after cerclage insertion (median 10 mm, range 1–30 mm for whole cohort). Women who did not demonstrate any increase in their CL or who actually displayed a reduction in CL (n = 21) were significantly more likely to deliver prior to 37 weeks gestation (57%) (RR 2.34, 95% CI 1.04–5.25), regardless of the degree of shortening prior to cerclage. This trend was also observed for RR of delivery prior to 34 weeks (33%), but did not reach statistical significance (Table 5).

**Table 5:** Risk of preterm birth by change in cervical length (mm) after cerclage insertion. The relative risk of preterm birth before 34 and 37 weeks gestation is shown in women who did not demonstrate cervical lengthening, or indeed shortened, after cerclage insertion, relative to those whose cervical lengths increased post cerclage procedure.

Gestation at delivery	Number of women with change in cervical length >0mm (n = 158)	Number of women with change in cervical length ≤0mm (n = 21)	RR (95% CI)
<34 weeks n (%)	38 (24%)	7 (33%)	1.49 (0.64–3.46) p = 0.36
<37 weeks n (%)	53 (34%)	12 (57%)	2.34 (1.04–5.25) p = 0.035

When change in CL following cerclage was treated as a continuous variable it was found that increasing CL did not reduce the odds of preterm delivery prior to 34 weeks (OR 0.99, 95% CI 0.94–1.03) or prior to 37 weeks (OR 0.98, 95% CI 0.93–1.02). This suggests the degree of lengthening is not relevant to outcome, but that the failure of cerclage to maintain the CL is associated with increased risk of PTB.

## Discussion

In this study, we described how women with a short cervix who had a cerclage placed in either the distal 10 mm or the distal half of the cervix were at greater risk of PTB compared with those with a higher placed stitch, and that increasing cerclage height reduced the odds of PTB at <34 and <37 weeks. We also assessed outcomes by CL at decision to suture and found, perhaps unsurprisingly, that increasing initial CL reduced the odds of subsequent PTB in this cohort. Following cerclage, we found that the failure of CL to return to ‘normal’ (>25mm) was associated with increased risk of PTB. Further shortening, or a static CL, were also associated with increased risk of early delivery.

There are limited data examining the anatomical location of USS indicated cerclage and subsequent pregnancy outcome. Rust et al. [11] studied 74 women who had a suture placed due to a CL <25mm or the presence of funnelling, and did not demonstrate a correlation between ‘cerclage position’ and gestational age at birth. However, this study included women with funnelling but a CL >25mm who would not have received a cerclage in our practice, and the authors defined total CL as the distance from the external os to the level of maximal funnel width, rather than the length of total closed cervix. Therefore, the potentially improved outcomes of many ‘high’ cerclages (>50% CL, >10mm to external os) could be combined with those with more superficial stitches resulting in a dilution of any improvement. Guzman et al. [12] assessed cerclage position in 29 women treated with emergency cerclage where the cervix was ≥2cm dilated. The authors found no correlation between proximity of cerclage to the internal os and gestation at delivery. However, the efficacy of ‘rescue cerclage’ compared with ‘USS indicated cerclage’ is much lower; in this study 48% delivered <36 weeks whilst in our cohort only 35% delivered <37 weeks gestation, which when combined with the small cohort size, may explain the differences in our findings.

Miroshnichenko et al. assessed cerclage position in 105 women undergoing cerclage secondary to poor obstetric history in the absence of cervical shortening [13]. Consistent with our findings, this study reported the incidence of spontaneous PTB reduced as the percentile height of cerclage increased. However, these results did not reach statistical significance, perhaps because of the smaller cohort and the reduced incidence of prematurity (16% delivered <35 weeks). Scheib et al. [14] examined a cohort of 70 women with USS indicated cerclage and found that a cerclage height of >18 mm is associated with a reduced risk of preterm delivery, which is consistent with our findings.

Our cohort was carefully characterised to include all TV USS indicated cerclages, excluding cerclages inserted secondary to poor obstetric history or to ‘rescue’ a pregnancy i.e. when the cervix is already dilated. Intra-observer variability was minimised by standardised scanning techniques and contemporaneous data collection. Close contact between the two units was maintained throughout the study period and only two senior obstetricians inserted all sutures thus reducing the potential impact of differing surgical technique.

Experience with ‘rescue’ cervical cerclage, and the rare closure of the cervix following the preterm delivery of only one twin, indicates cervical ripening and dilatation can, in some circumstances, be reversed [15]. The apparent lengthening of the cervix following cerclage is initially purely a mechanical effect. However, if this leads to a reversal of cervical ripening and the delivery is delayed, the neonatal outcome is likely to be improved. We examined how the change in CL following cerclage might predict outcome. We found in the minority of women whose CLs do not increase following cerclage, there is an increased risk of PTB (RR 2.34, 95% CI 1.04–5.25), and this effect was correspondingly less pronounced in women whose CLs simply remained ‘short’ (did not elongate above 25mm). This is probably a reflection of a failure to reverse the biochemical and physiological changes which had led to premature cervical ripening.

The exact mechanism by which cerclage helps to prevent or delay preterm labour is not entirely understood. It is likely that cerclage provides structural support to a weakened cervix, and enhances the cervical immunological barrier by improving retention of the mucous plug, and preventing ascending infection by maintaining CL. Cerclage placement may also reduce the extent of mechanical stretch at the level of the internal os. Our finding that women with more superficially placed cerclages are at greater risk of PTB is in keeping with the apparent high success of abdominal cerclage, even in very high-risk women, where insertion is at the cervicoisthmic junction [16].

Preterm birth is a heterogeneous syndrome with multiple causes. To date, with the exception of multiple pregnancy [17], no trial of any predictive tool or intervention has attempted to stratify subjects into aetiological subgroups. Use of ultrasound measurements of CL, or biomarkers such as fibronectin, help to define a higher risk population, but since cervical shortening and release of fibronectin are likely to precede preterm birth over a range of aetiologies, their ability to predict which women would benefit from cerclage is limited. Our cohort will, of course, reflect this diversity and women should be informed that cerclage may prolong their pregnancy, but there are some associated risks. Intraoperative complications including bladder damage, cervical trauma, membrane rupture and bleeding are rare and are reported as occurring in <1% of cases.

We have reported on the largest cohort of USS-indicated cervical cerclage to date and shown that the anatomical location of suture placement within the cervix is correlated with subsequent risk of PTB. We recommend every effort should be made to place a cerclage as high as possible. Women whose CL fails to increase, or shortens further following cerclage placement, should remain under close clinical surveillance as this group is most at risk of PTB (RR 2.34).

## References

[1] Shennan A, Jones B. The cervix and prematurity: aetiology, prediction and prevention. Seminars in fetal & neonatal medicine. 2004 12;9[6]:471–9. 15691785 10.1016/j.siny.2004.09.001

[2] Berghella V, Rafael TJ, Szychowski JM, Rust OA, Owen J. Cerclage for short cervix on ultrasonography in women with singleton gestations and previous preterm birth: a meta-analysis. Obstetrics and gynecology. 2011 3;117[3]:663–71. 21446209 10.1097/AOG.0b013e31820ca847

[3] Berghella V, Odibo AO, Tolosa JE. Cerclage for prevention of preterm birth in women with a short cervix found on transvaginal ultrasound examination: a randomized trial. American journal of obstetrics and gynecology. 2004 10;191[4]:1311–7. doi: 10.1016/j.ajog.2004.06.054 15507959 10.1016/j.ajog.2004.06.054

[4] Alfirevic Z, Stampalija T, Roberts D, Jorgensen AL. Cervical stitch (cerclage) for preventing preterm birth in singleton pregnancy. The Cochrane database of systematic reviews. 2012;4:CD008991. 10.1002/14651858.CD008991.pub2 22513970

[5] To MS, Alfirevic Z, Heath VC, Cicero S, Cacho AM, Williamson PR, et al Cervical cerclage for prevention of preterm delivery in women with short cervix: randomised controlled trial. Lancet. 2004 6 5;363[9424]:1849–53. 15183621 10.1016/S0140-6736(04)16351-4

[6] McDonald IA. Suture of the cervix for inevitable miscarriage. The Journal of obstetrics and gynaecology of the British Empire. 1957 6;64[3]:346–50. 13449654 10.1111/j.1471-0528.1957.tb02650.x

[7] Berghella V, Ness A, Bega G, Berghella M. Cervical sonography in women with symptoms of preterm labor. Obstetrics and gynecology clinics of North America. 2005 9;32[3]:383–96. Epub 2005/08/30. eng. doi: 10.1016/j.ogc.2005.04.007 16125039 10.1016/j.ogc.2005.04.007

[8] Mella MT, Berghella V. Prediction of preterm birth: cervical sonography. Seminars in perinatology. 2009 10;33[5]:317–24. doi: 10.1053/j.semperi.2009.06.007 19796729 10.1053/j.semperi.2009.06.007

[9] Breiman L., Friedman J. H., Olshen R. A., and Stone, C. J. (1984) Classification and Regression Trees. Wadsworth

[10] Alabi-Isama L, Sykes L, Chandiramani M, Patel S, Rai R, Bennett PR, et al Time interval from elective removal of cervical cerclage to onset of spontaneous labour. European journal of obstetrics, gynecology, and reproductive biology. 2012 12;165[2]:235–8. doi: 10.1016/j.ejogrb.2012.09.005 23018099 10.1016/j.ejogrb.2012.09.005

[11] Rust OA, Atlas RO, Meyn J, Wells M, Kimmel S. Does cerclage location influence perinatal outcome? American journal of obstetrics and gynecology. 2003 12;189[6]:1688–91. 14710099 10.1016/s0002-9378(03)00779-8

[12] Guzman ER, Houlihan C, Vintzileos A, Ivan J, Benito C, Kappy K. The significance of transvaginal ultrasonographic evaluation of the cervix in women treated with emergency cerclage. American journal of obstetrics and gynecology. 1996 8;175[2]:471–6. 8765271 10.1016/s0002-9378(96)70164-3

[13] Miroshnichenko G, Visintine JF, Suhag A, Gerson A, Berghella V. Is cerclage height associated with the incidence of preterm birth in women with a history-indicated cerclage? American journal of perinatology. 2011 1;28[1]:83–6. doi: 10.1055/s-0030-1262908 20640973 10.1055/s-0030-1262908

[14] Scheib S, Visintine JF, Miroshnichenko G, Harvey C, Rychlak K, Berghella V. Is cerclage height associated with the incidence of preterm birth in women with an ultrasound-indicated cerclage? American journal of obstetrics and gynecology. 2009 5;200[5]:e12–5. doi: 10.1016/j.ajog.2008.09.021 19026400 10.1016/j.ajog.2008.09.021

[15] Reinhard J, Reichenbach L, Ernst T, Reitter A, Antwerpen I, Herrmann E, et al Delayed interval delivery in twin and triplet pregnancies: 6 years of experience in one perinatal center. Journal of perinatal medicine. 2012 9;40[5]:551–5. doi: 10.1515/jpm-2011-0267 23104798 10.1515/jpm-2011-0267

[16] Fick A.L., Caughey A.B. & Parer J.T. Transabdominal cerclage: can we predict who fails? The journal of maternal-fetal & neonatal medicine: the official journal of the European Association of Perinatal Medicine, the Federation of Asia and Oceania Perinatal Societies, the International Society of Perinatal Obstet 20, 63–67 [2007]. 10.1080/14767050601059156 17437202

[17] Berghella V, Odibo AO, To MS, Rust OA, Althuisius SM. Cerclage for short cervix on ultrasonography: meta-analysis of trials using individual patient-level data. Obstetrics and gynecology. 2005 7;106[1]:181–9. doi: 10.1097/01.AOG.0000168435.17200.53 15994635 10.1097/01.AOG.0000168435.17200.53

